# Clinical Characteristics and Long-Term Outcomes of Late-Onset Multiple Sclerosis: A Swedish Nationwide Study

**DOI:** 10.1212/WNL.0000000000208051

**Published:** 2024-02-23

**Authors:** Elena F. Mouresan, Eleni Mentesidou, Anders Berglund, Kyla A. McKay, Jan Hillert, Ellen Iacobaeus

**Affiliations:** From the Department of Clinical Neuroscience (E.F.M., E.M., A.B., K.A.M., J.H., E.I.), and Centre for Molecular Medicine (J.H.), Karolinska Institutet; and Department of Neurology (E.M., K.A.M., E.I.), Stockholm, Sweden.

## Abstract

**Background and Objectives:**

Clinical onset of multiple sclerosis (MS) after the age of 50 years is uncommon and associated with a less favorable natural history. The differences in long-term outcomes in patients with late-onset MS (LOMS, onset 50 years or older) and adult-onset MS (AOMS, onset 18 years or older and younger than 50 years) during the disease-modifying therapy (DMT) era have been less studied. This study aimed to compare patient characteristics, DMT exposure, and disability progression in Swedish patients with LOMS and AOMS over 2 decades (2001–2022).

**Methods:**

The nationwide Swedish MS registry was searched for patients with an onset of MS between January 1, 2001, and December 31, 2018, with symptom onset at age 18 years or older and ≥2 recorded Expanded Disability Status Scale (EDSS) scores. Clinical and demographic parameters and exposure to DMT were compared between LOMS and AOMS. Time to disability milestones (EDSS 4 and 6) was assessed using Kaplan-Meier curves and Cox proportional hazards regression models adjusted for sex, disease course, calendar year at onset, and DMT exposure.

**Results:**

Among 8739 patients with MS who met inclusion criteria, 1,028 (11.8%) were LOMS. Primary progressive MS was more frequently diagnosed in LOMS compared with that in AOMS (25.2% vs 4.5%; *p* < 0.001). Most of the patients had been prescribed DMT, but more rarely in LOMS compared with AOMS (74.7% vs 95.6%; *p* < 0.001). Less than half of patients with LOMS had been exposed to a high-efficacy DMT (45.8%) compared with 73.5% of AOMS (*p* < 0.001). The risk of reaching disability milestones was greater for LOMS compared with that for AOMS (EDSS 4; adjusted hazard ratio [aHR] 2.71; 95% CI 2.22–3.30; *p* < 0.001, and EDSS 6; aHR 2.67; 95% CI 2.12–3.36; *p* < 0.001).

**Discussion:**

This study distinguishes LOMS as a particularly vulnerable group and clinically supports close vigilance of these patients. Further studies are needed to assess and clarify the benefit of DMT usage in older adults with MS.

## Introduction

Multiple sclerosis (MS) is an inflammatory demyelinating disease of the CNS that typically effects young adults. However, a subgroup of patients present with “late-onset MS” (LOMS), in which their symptoms begin at 50 years of age or later.^1^

Old age has been identified as an independent parameter for an unfavorable disease evolution in MS.^2-5^ Factors that can contribute to this worse prognosis include an increased prevalence of comorbidities, diminished exposure and efficacy of disease-modifying therapies (DMTs), age-associated neurodegenerative processes, and immune senescence.^6^

With increased longevity in the general population, a rising incidence and prevalence of LOMS is expected. In fact, a growing aged MS population has been observed in data from real-world studies.^7,8^ A recent US claims data analysis of 125 million adults described a peak prevalence of MS between 55 and 64 years of age.^9^ Furthermore, an increasing prevalence of MS, including LOMS, has been identified during the latest decade.^10-12^ Due to the exclusion of older adults from most clinical trials, there is limited knowledge about DMT efficacy and safety in patients with LOMS. In this study, we aimed to compare clinical characteristics, DMT use, and disability progression in persons with LOMS and adult-onset MS (AOMS, onset between ages 18 and 49 years) using Swedish registry data.

## Methods

### Study Design, Demography, and Clinical Characteristics of the Patients

This was a nationwide cohort study that used the Swedish MS registry (SMSreg) as data source. The registry contains individual patient information that was prospectively recorded from neurology clinics across Sweden starting from 2000. It is estimated to capture approximately 85% of all prevalent cases of MS in the population with nationwide coverage.^13^ Participation is voluntary with patients providing consent for their data to be used for clinical and research purposes. Data used in this study were extracted on January 27, 2022. Inclusion criteria comprised a definite diagnosis of MS and year of symptom onset between January 1, 2001, and December 31, 2018, to allow sufficient follow-up time before the study end date of December 31, 2021. Those with an age at year of symptom onset younger than 18 years and patients with <2 Expanded Disability Status Scale (EDSS)^14^ scores recorded in the SMSreg were excluded. Persons were categorized as LOMS if symptom onset occurred at 50 years or older and AOMS if symptom onset was at 18 years or older and at younger than 50 years.

The clinical and demographic information extracted from the SMSreg for each patient included sex, date of birth, date of MS onset and diagnosis, date and reason for withdrawal from the registry (i.e., death or emigration), clinical course, information on disease-modifying therapy (DMT) use (product name, start and end dates), and date of visit to the neurologist with EDSS measurements recorded. The definition “onset of MS” referred to the time of the first appearance of MS symptoms. The diagnostic delay was calculated as the time from MS onset to diagnosis. The total follow-up time was calculated as the time from MS onset to the date of death, withdrawal from the registry, or the date of the study end (whichever came first). Exposure to DMT was categorized as none (no DMT use recorded), modest-efficacy therapy only (interferon beta, glatiramer acetate, teriflunomide, and dimethyl fumarate), high-efficacy therapy only (natalizumab, rituximab, ocrelizumab, fingolimod, alemtuzumab, cladribine, daclizumab, and autologous hematopoietic stem cell transplantation (aHSCT), or both (exposure to both modest-efficacy and high-efficacy DMT). EDSS score at diagnosis was defined as the earliest EDSS measurement recorded within a year from MS diagnosis.

### Outcome Measures

Disability progression over time was assessed by measuring the time from MS onset to confirmed sustained EDSS disability milestones 4 and 6. An event of disability progression was defined when a patient had 2 consecutive visits at least 150 days apart with an EDSS score of ≥4 or ≥6 (confirmed) and no future EDSS score <4 or <6 (sustained). The date of the initial worsening was selected as the time point of the event.

### Statistical Analysis

Descriptive demographic and clinical characteristics of the cohort and in disease subtypes (relapsing-remitting MS [RRMS] and primary progressive MS [PPMS]) were reported as medians and interquartile range (IQR) for numerical variables and number and percentages for categorical variables. The characteristics were compared between LOMS and AOMS cohorts using the Pearson χ^2^ test for categorical variables and the Wilcoxon rank sum test for continuous variables.

Nonparametric Kaplan-Meier estimators were applied to assess the likelihood of reaching confirmed sustained EDSS 4 and 6. The difference in rates of reaching EDSS 4 and 6 according to age at onset (LOMS vs AOMS) was assessed using Cox proportional hazards (Cox PH) regression analysis. Multivariable models were adjusted for potential confounders including sex, disease course at onset (RRMS/PPMS), calendar year at onset, and DMT exposure (no treatment/modest efficacy only/high efficacy only/both modest and high efficacy). In Cox PH models that did not fulfill the proportionality assumption, a parametric model (Weibull) was applied instead. Variables that did not fulfill the proportionality assumption in the multivariable Cox PH model were stratified (calendar year).

Second, univariable and multivariable Cox regression analyses were used to assess the influence of clinical and demographic characteristics, including age at onset, sex, disease course, calendar year at onset, and treatment exposure, on the risk of reaching EDSS 4 and 6 in the LOMS cohort. Statistical analyses were performed using R: A language and environment for statistical computing (R Foundation for Statistical Computing, Vienna, Austria) V.4.2.1.

### Standard Protocol Approvals, Registrations, and Patient Consents

This study was approved by the Swedish Ethical Review Authority (Dnr 2022-00684-01). Study consent was provided from the patients at first inclusion in the SMSreg; the consent covers all research based on data from the registry, and further acquisition of informed consent is not required.

### Data Availability

For data sharing from the SMSreg, a data transfer agreement is required, in accordance with the data protection legislation in Europe (General Data Protection Regulation). In the matter of interest in obtaining data access, please contact Jan Hillert (jan.hillert@ki.se).

## Results

### Clinical Characteristics of the Study Patients

**Figure 1:**
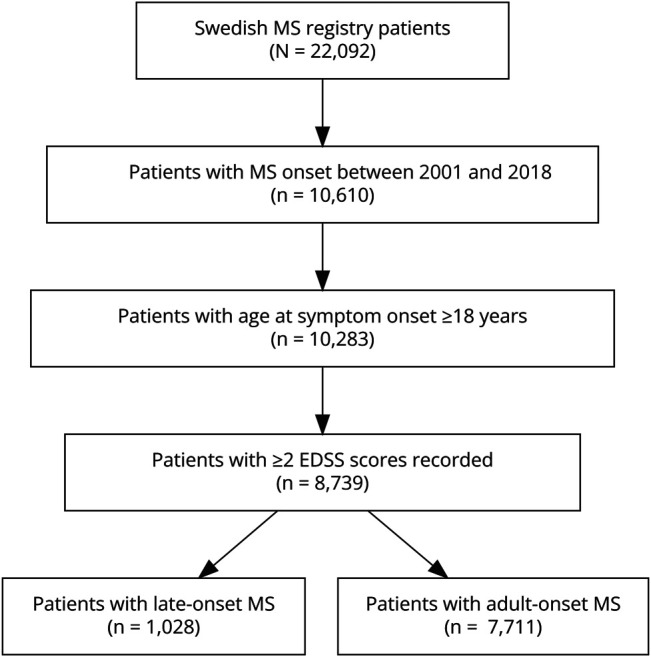
Flowchart of Patient Inclusion EDSS = Expanded Disability Status Scale; MS = multiple sclerosis.

As of December 31, 2021, a total of 10,610 patients had incident MS during the study period. Among these individuals, 8,739 (82.4%) met inclusion criteria, including 1,028 (11.8%) cases with LOMS and 7,711 (88.2%) cases with AOMS. Schematic information about the inclusion of patients is provided in Figure 1. Table 1 describes the demographic and clinical details of the cohort. The median age at disease onset in LOMS was 54 years (IQR: 52–58 years) and 32 years (IQR: 26–39 years) in AOMS, while the median age at diagnosis was 56 years (IQR: 53–60 years) in LOMS and 34 years (IQR: 28–41 years) in AOMS. Higher disability scores were found in LOMS at diagnosis (median EDSS: 2.5, IQR: 1.5–3.5) compared with those in AOMS (median EDSS: 1.5, IQR: 1.0–2.5, *p* < 0.001).

**Table 1:** Demographic and Clinical Characteristics of the Study Populations

	Overall			RRMS			PPMS		
	LOMS (N = 1,028)	AOMS (N = 7,711)	*p* Value	LOMS (N = 753)	AOMS (N = 7,283)	*p* Value	LOMS (N = 254)	AOMS (N = 344)	*p* Value
Age at onset, median (IQR)	54.0 (52.0–58.0)	32.0 (26.0–39.0)	<0.001^a^	54.0 (51.0–57.0)	32.0 (26.0–39.0)	<0.001^a^	55.0 (52.0–59.0)	42.0 (35.0–46.0)	<0.001^a^
Age at diagnosis, median (IQR)	56.0 (53.0–60.0)	34.0 (28.0–41.0)	<0.001^a^	55.0 (53.0–59.0)	33.0 (27.0–41.0)	<0.001^a^	58.0 (55.0–62.0)	45.0 (38.0–49.0)	<0.001^a^
Diagnostic delay, years median (IQR)	1.0 (0.0–3.0)	1.0 (0.0–2.0)	<0.001^a^	1.0 (0.0–2.0)	1.0 (0.0–2.0)	0.2^a^	2.0 (1.0–4.0)	2.0 (1.0–5.0)	0.037^a^
Follow-up duration, years median (IQR)	11.5 (7.7–15.7)	11.9 (8.0–16.3)	0.042^a^	11.0 (7.2–15.5)	11.8 (7.8–16.2)	0.004^a^	12.0 (8.8–16.5)	13.8 (10.5–17.4)	0.004^a^
Sex, n (%)			0.048^b^			0.3^b^			0.041^b^
Female	675 (65.7)	5,298 (68.7)		509 (67.6)	5,063 (69.5)		147 (57.9)	174 (50.6)	
Male	353 (34.3)	2,412 (31.3)		244 (32.4)	2,219 (30.5)		107 (42.1)	170 (49.4)	
Initial disease course, n (%)			<0.001^b^			—			—
RRMS	753 (74.8)	7,283 (95.5)		753 (100.0)	7,283 (100.0)		0	0	
PPMS	254 (25.2)	344 (4.5)		0	0		254 (100.0)	344 (100.0)	
Total EDSS scores, median (IQR)	6.0 (3.0–9.0)	8.0 (5.0–11.0)	<0.001^a^	6.0 (4.0–9.0)	8.0 (5.0–12.0)	<0.001^a^	5.0 (3.0–7.8)	6.0 (3.0–9.0)	<0.001^a^
EDSS at diagnosis, median (IQR)	2.5 (1.5–3.5)	1.5 (1.0–2.5)	<0.001^a^	2.0 (1.0–3.0)	1.5 (1.0–2.5)	<0.001^a^	3.5 (2.5–5.0)	3.0 (2.5–4.5)	0.036^a^
DMT exposure, n (%)			<0.001^b^			<0.001^b^			<0.001^b^
None	260 (25.3)	336 (4.4)		96 (12.7)	209 (2.9)		153 (60.2)	103 (29.9)	
Modest	297 (28.9)	1,704 (22.1)		273 (36.3)	1,633 (22.4)		18 (7.1)	41 (11.9)	
High	257 (25.0)	2,014 (26.1)		181 (24.0)	1,863 (25.6)		74 (29.1)	144 (41.9)	
Both	214 (20.8)	3,657 (47.4)		203 (27.0)	3,578 (49.1)		9 (3.5)	56 (16.3)	

Abbreviations: AOMS = adult-onset multiple sclerosis; DMT = disease-modifying therapy; EDSS = Expanded Disability Status Scale; IQR = interquartile range; LOMS = late-onset multiple sclerosis; PPMS = primary progressive multiple sclerosis; RRMS = relapsing-remitting multiple sclerosis.

Modest (efficacy) DMT: interferon beta-1a, interferon beta-1b, pegylated interferon beta-1a, glatiramer acetate, dimethyl fumarate, and teriflunomide.

High (efficacy) DMT: rituximab, ocrelizumab, natalizumab, alemtuzumab, fingolimod, cladribine, daclizumab, and autologous hematopoietic stem cell transplantation.

a Wilcoxon rank sum test.

b Chi-square test.

Progressive onset was more prevalent in LOMS (25.2%) compared with that in AOMS (4.5%). A female predominance was found in both groups, but a lower proportion of LOMS were female (65.7% vs 68.7%, *p* < 0.05).

### Exposure to Disease Modifying Therapies in LOMS

**Figure 2:**
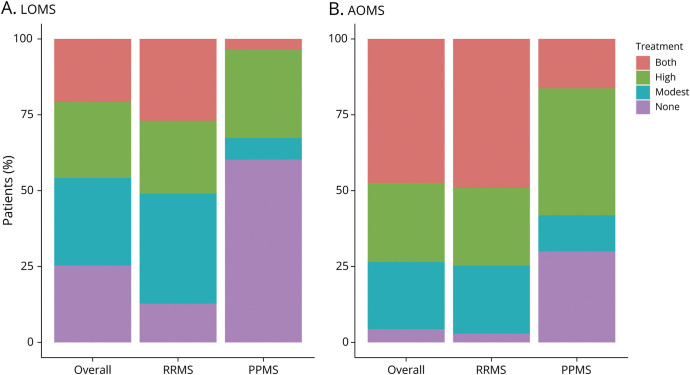
Treatment Exposure in the Study Population (A) Late-onset multiple sclerosis (LOMS); (B) Adult-onset multiple sclerosis. PPMS = primary progressive multiple sclerosis; RRMS = relapsing-remitting multiple sclerosis. Modest (efficacy) DMT: interferon beta-1a, interferon beta-1b, pegylated interferon beta-1a, glatiramer acetate, dimethyl fumarate, and teriflunomide. High (efficacy) DMT: rituximab, ocrelizumab, natalizumab, alemtuzumab, fingolimod, cladribine, daclizumab, and autologous hematopoietic stem cell transplantation.

LOMS were less frequently prescribed DMTs compared with AOMS (74.7% vs 95.6%; *p* < 0.001) (Table 1 and Figure 2). While exposure to only modest-efficacy or only high-efficacy DMTs was comparable between the cohorts (LOMS-modest 28.9%/LOMS-high 25.0% vs AOMS-modest 22.1%/AOMS-high 26.1%), patients with LOMS were less likely to switch between modest-efficacy and high-efficacy DMTs (LOMS 20.8% vs AOMS 47.4%, *p* < 0.001), and exposure to high-efficacy DMTs was less frequent in LOMS compared with that in AOMS (LOMS 45.8% vs AOMS 73.5%, *p* < 0.001). Subgroup analysis for initial disease course showed that most of the patients with RRMS had received a DMT (87.3% RR-LOMS and 97.1% RR-AOMS, *p* < 0.001) compared with 39.8% of LOMS and 70.1% of AOMS (*p* < 0.001) with progressive onset. High-efficacy therapy was initiated in 51.0% of RR-LOMS and 74.7% of RR-AOMS compared with approximately one-third (32.6%) of PP-LOMS and 58.2% of PP-AOMS.

### Disability Progression

**Figure 3:**
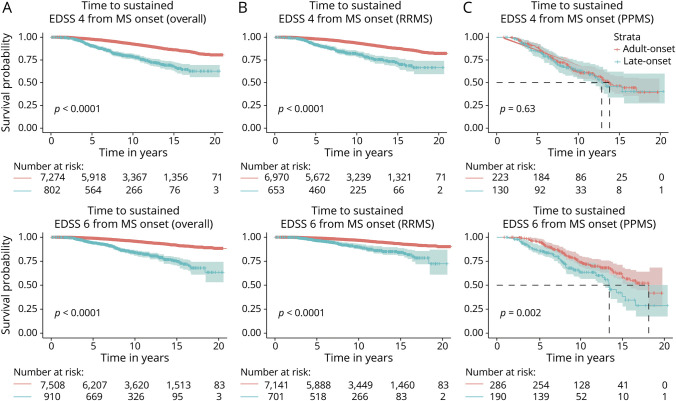
Kaplan-Meier Curves of Time to Confirmed Sustained EDSS 4 and 6 Total cohort (A), RRMS subcohort (B), PPMS subcohort (C). AOMS = adult-onset multiple sclerosis; LOMS = late-onset multiple sclerosis; PPMS = primary progressive multiple sclerosis; RRMS = relapsing-remitting multiple sclerosis.

The median time to reach both disability milestones was significantly shorter for LOMS than AOMS (Figure 3). Similarly, in the analyses of RRMS, the LOMS experienced faster disability progression (Figure 3), but in the analyses of PPMS, there was only a significantly shorter time to EDSS 6, but not 4, among the LOMS (Figure 3).

In the adjusted analyses, the risk of reaching EDSS 4 and 6 was higher among patients with LOMS compared with patients with AOMS (EDSS 4: aHR 2.71; 95% CI 2.22–3.30; EDSS 6: aHR 2.67; 95% CI 2.12–3.36) (Table 2). While the risk of patients with RR-LOMS was 3 times higher than that of patients with RR-AOMS for both milestones, the risk for patients with progressive LOMS was similar to that for patients with PP-AOMS for the EDSS 4 milestone (aHR 1.11; 95% CI 0.75–1.65) and 67% higher for EDSS 6 (aHR 1.67; 95% CI 1.16–2.41) (Table 2).

**Table 2:** Risk of Reaching Disability Milestones From MS Onset in LOMS Compared With That in AOMS

	Overall		RRMS		PPMS	
	HR (95% CI)	Adjusted HR^a^ (95% CI)	HR (95% CI)	Adjusted HR^a^ (95% CI)	HR (95% CI)	Adjusted HR^a^ (95% CI)
From MS onset to						
EDSS 4	3.11^b^ (2.61–3.71)	2.71^c^ (2.22–3.30)	3.04^b^ (2.47–3.73)	3.69^b^ (2.97–4.58)	1.09^c^ (0.76–1.57)	1.11^c^ (0.75–1.65)
EDSS 6	3.80^c^ (3.11–4.64)	2.67^c^ (2.12–3.36)	2.99^c^ (2.28–3.91)	3.61^c^ (2.72–4.79)	1.68^c^ (1.20–2.33)	1.67^c^ (1.16–2.41)

Abbreviations: CI = confidence interval; EDSS = Expanded Disability Status Scale; MS = multiple sclerosis; PPMS = primary progressive multiple sclerosis; RRMS = relapsing-remitting multiple sclerosis.

a Adjusted for sex, calendar year at MS onset, DMT exposure (none, both, modest, or high efficay) and initial disease course (RRMS or PPMS).

b Weibull model.

c Cox PH model.

Results from the univariable models within the LOMS cohort revealed that higher age at onset and PPMS course were associated with a faster time to EDSS 6, while calendar year at onset and exposure to DMT were associated with slower time to EDSS 6 (Table 3). The multivariable analysis revealed that neither sex, calendar year at onset, nor DMT exposure influenced time to disability milestones within the LOMS cohort. Only PPMS disease course had a significant association with progression (EDSS 4: 2.42, 95% CI 1.59–3.68 and EDSS 6: 4.14, 95% CI 2.66–6.45) (Table 4).

**Table 3:** Univariate Cox Models of Time to EDSS 4 and 6 in LOMS

Characteristic	EDSS 4			EDSS 6		
	Overall (N = 802)	RRMS (N = 653)	PPMS (N = 130)	Overall (N = 910)	RRMS (N = 701)	PPMS (N = 190)
	HR (95% CI)	HR (95% CI)	HR (95% CI)	HR (95% CI)	HR (95% CI)	HR (95% CI)
Age at onset	1.02 (0.98–1.06)	1.02 (0.97–1.07)	0.99 (0.93–1.06)	1.06 (1.02–1.10)	1.05 (0.99–1.11)	1.03 (0.97–1.08)
Sex						
Female	1.0 (−)	1.0 (−)	1.0 (−)	1.0 (−)	1.0 (−)	1.0 (−)
Male	1.18 (0.85–1.65)	1.29 (0.87–1.91)	0.74 (0.40–1.38)	1.13 (0.79–1.62)	1.04 (0.62–1.76)	0.99 (0.59–1.64)
Initial course						
RRMS	1.0 (−)	—	—	1.0 (−)	—	—
PPMS	2.26 (1.59–3.20)	—	—	4.20 (2.97–5.94)	—	—
Calendar year at MS onset	0.97 (0.94–1.01)	0.99 (0.95–1.04)	0.95 (0.88–1.02)	0.95 (0.91–0.99)	0.97 (0.91–1.04)	0.95 (0.89–1.02)
DMT exposure						
None	1.0 (−)	1.0 (−)	1.0 (−)	1.0 (−)	1.0 (−)	1.0 (−)
Modest	0.96 (0.62–1.47)	1.65 (0.83–3.25)	1.63 (0.73–3.66)	0.50 (0.33–0.76)	1.53 (0.67–3.48)	1.22 (0.55–2.73)
High	1.14 (0.69–1.90)	1.84 (0.83–4.04)	0.87 (0.42–1.79)	0.68 (0.41–1.14)	1.58 (0.58–4.30)	0.64 (0.34–1.21)
Both	0.90 (0.56–1.44)	1.61 (0.80–3.26)	0.93 (0.23–3.95)	0.43 (0.27–0.76)	1.39 (0.59–3.30)	0.61 (0.15–2.52)

Abbreviations: CI = confidence interval; DMT = disease-modifying therapy; EDSS = Expanded Disability Status Scale; PPMS = primary progressive multiple sclerosis; RRMS = relapsing-remitting multiple sclerosis.

Modest (efficacy) DMT: interferon beta-1a, interferon beta-1b, peginterferon, glatiramer acetate, dimethyl fumarate, teriflunomide.

High (efficacy) DMT: rituximab, ocrelizumab, natalizumab, alemtuzumab, fingolimod, cladribine, daclizumab, and hematopoietic stem cell transplantation.

**Table 4:** Multivariate Cox Models of Time to EDSS 4 and 6 in LOMS

	EDSS 4			EDSS 6		
	Overall (N = 783)	RRMS (N = 653)	PPMS (N = 130)	Overall (N = 891)	RRMS (N = 701)	PPMS (N = 190)
	HR (95% CI)	HR (95% CI)	HR (95% CI)	HR (95% CI)	HR (95% CI)	HR (95% CI)
Age at onset	1.02 (0.98–1.06)	1.03 (0.98–1.08)	0.98 (0.91–1.06)	1.04 (0.99–1.08)	1.05 (1.00–1.13)	1.02 (0.97–1.08)
Sex						
Female	1.0 (−)	1.0 (−)	1.0 (−)	1.0 (−)	1.0 (−)	1.0 (−)
Male	1.10 (0.78–1.54)	1.24 (0.84–1.85)	0.73 (0.39–1.40)	1.05 (0.73–1.51)	0.99 (0.58–1.69)	1.02 (0.60–1.73)
Initial course						
RRMS	1.0 (−)	—	—	1.0 (−)	—	—
PPMS	2.42 (1.59–3.68)	—	—	4.14 (2.66–6.45)	—	—
Calendar year at MS onset	1.97 (0.93–1.02)	0.97 (0.92–1.02)	0.96 (0.89–1.04)	0.96 (0.91–1.01)	0.95 (0.88–1.02)	0.96 (0.89–1.03)
DMT exposure						
None	1.0 (−)	1.0 (−)	1.0 (−)	1.0 (−)	1.0 (−)	1.0 (−)
Modest	1.43 (0.88–2.33)	1.73 (0.87–3.45)	1.54 (0.68–3.53)	1.16 (0.70–1.93)	1.77 (0.77–4.11)	1.15 (0.50–2.60)
High	1.42 (0.82–2.47)	2.28 (0.95–5.47)	0.91 (0.42–1.97)	1.04 (0.60–1.80)	2.56 (0.83–7.92)	0.74 (0.37–1.46)
Both	1.41 (0.82–2.44)	1.80 (0.86–3.45)	0.74 (0.16–3.45)	1.12 (0.61–2.04)	1.88 (0.75–4.72)	0.68 (0.16–2.96)

Abbreviations: CI = confidence interval; DMT = disease-modifying therapy; EDSS = Expanded Disability Status Scale; PPMS = primary progressive multiple sclerosis; RRMS = relapsing-remitting multiple sclerosis.

Modest (efficacy) DMT: interferon beta-1a, interferon beta-1b, pegylated interferon beta-1a, glatiramer acetate, dimethyl fumarate, and teriflunomide.

High (efficacy) DMT: rituximab, ocrelizumab, natalizumab, alemtuzumab, fingolimod, cladribine daclizumab, and autologous hematopoietic stem cell transplantantation.

## Discussion

This large nationwide study found that nearly 12% of incident cases of MS begin after the age of 50 years and that these persons experienced more rapid disability progression than the more common adult-onset MS. Notable factors distinguished LOMS including an increased prevalence of progressive disease course, less exposure to DMTs, even among relapsing-remitting patients, and limited benefit of DMTs to halt disease progression.

Progressive-onset MS was more frequent in LOMS compared with that in AOMS, which is in line with previous studies, in which estimates ranged from 25.6% to 54.5%,^15-19^ and the natural history of PPMS, which is known to have a later mean onset age. Our results were stratified by disease subtype with consistencies across both groups, namely the worse disability progression seen among LOMS and the reduced use of DMTs. However, the higher proportion of male individuals and shorter diagnostic delay in LOMS were only different in the PPMS subcohorts. While there were more women in all subgroups studied, a higher proportion of men characterized LOMS and was particularly strong in the PP-LOMS.

LOMS exhibited faster disability progression compared with AOMS, but disability milestones were reached later in life, because of the later age at onset. These findings are consistent with results from 3 earlier studies.^15,16,18^ Outcome analyses based on disease type at onset showed that the differences between LOMS and AOMS were greatest in the RRMS cohort, with upward of 3 times the probability of reaching disability milestones. On the contrary, PPMS-LOMS conferred the same risk to reach EDSS 4.0 but increased risk to reach EDSS 6.0, compared with PP-AOMS. Taken together, our observations indicated stronger similarities between PPMS across LOMS and AOMS, compared with RRMS, underlining the need for subgroup analyses on disease subtype in future LOMS studies. Approximately 75% of patients with LOMS had received treatment in this study, which is comparable with a recent French study that reported prescription of DMTs in 65.3% of LOMS.^16^

The high frequency of modest-efficacy DMT prescription was in line with previous findings and likely due to their established safety profile, with particular importance in older adults who frequently bear a higher occurrence of comorbidities and pharmacotherapy usage.^20^ Furthermore, in both disease subtypes, the LOMS group had a much higher proportion of persons never treated with a DMT.

In adjusted models, we found no evidence for a beneficial effect of DMT on longer-term disability in LOMS. Knowledge regarding DMT efficacy in LOMS is scarce. An Italian registry study investigated long-term outcomes in LOMS exposed to injectables (interferons and glatiramer acetate) vs oral agents (dimethyl fumarate and teriflunomide) and demonstrated a low risk for disability progression, during a mean follow-up time of 25.8 months (31.6% in the injectable group and 18.7% in the oral group).^21^ Less encouraging results were described in studies on subgroups of patients with MS initiating high-efficacy DMTs at older ages; rituximab,^22^ natalizumab, and fingolimod^23,24^ failed to prevent disease progression. The interpretation of study results of older patients with MS whose disease may have started decades earlier to LOMS populations should, however, be performed with caution considering the difference in disease duration. Additional studies focusing on DMT use, specifically among LOMS, are needed to gain more insight into their longer-term safety and effectiveness.

Several lines of evidence have postulated that neurodegenerative processes are the main driver for disability progression in MS, which indicate that age-associated neuronal damage plays an important role for the worse prognosis in LOMS.^25^ Older age is associated with limited recovery from relapses and greater loss of whole brain volume.^26,27^ Age-related cell defects in the CNS drive neurodegenerative processes through heightened oxidative stress level, impaired DNA repair, genomic aberrations, and triggering of proinflammatory processes.^28^ Additional factors with relevance for the aging brain include an increased frequency of comorbidities such as cardiovascular disease, metabolic changes, and reproductive senescence.^29-31^ Furthermore, findings suggesting a preaged immune system in patients with MS have been detected. Patients with MS older than 60 years exerted reduced capacity of coinhibitory immune cell signaling and prominent increase in the levels of circulating cytotoxic CD4^+^ T cells, which play a role in compartmentalized CNS inflammation in progressive MS.^32,33^

A limitation of this study is the difference in follow-up between groups, such that patients with the highest disability scores, in addition to those with the oldest age, more often are lost to follow-up.^34^ Although this is present in both LOMS and AOMS, it is possible that the LOMS group included a greater proportion of such patients and therefore may have affected the current findings. Furthermore, we lacked information on adherence, and we did not account for confounding by indication, so we cannot draw conclusions regarding DMT effectiveness.

The approach to MS pharmacologic treatment has shifted over the past 2 decades in Sweden. Initially, an escalation approach was adopted, in which modest-efficacy DMTs were used as first treatments and switched to a high-efficacy DMT at breakthrough disease. In later years, an increasing proportion of patients have been prescribed high-efficacy DMTs as the first treatment choice, in line with revised national treatment guidelines.^35^ Furthermore, there are no explicit restrictions for DMT prescription in relation to old age, disability (high EDSS), or progressive MS for which individual treatment decisions rather are applied. The current Swedish treatment guidelines are thereby different from other European and American guidelines that may limit translation of our findings.^36,37^ At last, we did not have information on comorbidities, which are more common among older adults and associated with a worse prognosis in MS,^38^ and qualify for a separate study to validate our findings.

LOMS accounts for nearly 12% of the MS population and is associated with a worse prognosis than AOMS. The increased risk for rapid disability progression in LOMS was independent of DMT exposure and found in both disease subtypes. Our study highlights the challenges of treating older adults with MS in the era of highly-effective DMTs. Clinical trials that include a wider age range and real-world data on DMTs will be needed to optimize treatment and care of LOMS.

## Appendix Authors

**Table:**

Name	Location	Contribution
Elena F. Mouresan, MD	Department of Clinical Neuroscience, Karolinska Institutet, Stockholm, Sweden	Drafting/revision of the article for content, including medical writing for content; major role in the acquisition of data; study concept or design; and analysis or interpretation of data
Eleni Mentesidou, MD	Department of Clinical Neuroscience, Karolinska Institutet; Department of Neurology, Karolinska University Hospital, Stockholm, Sweden	Drafting/revision of the article for content, including medical writing for content; major role in the acquisition of data; study concept or design; and analysis or interpretation of data
Anders Berglund, MD	Department of Clinical Neuroscience, Karolinska Institutet, Stockholm, Sweden	Drafting/revision of the article for content, including medical writing for content; major role in the acquisition of data; study concept or design; and analysis or interpretation of data
Kyla A. McKay, MD	Department of Clinical Neuroscience, Karolinska Institutet; Department of Neurology, Karolinska University Hospital, Stockholm, Sweden	Drafting/revision of the article for content, including medical writing for content; study concept or design; and analysis or interpretation of data
Jan Hillert, MD, PhD	Department of Clinical Neuroscience; Centre for Molecular Medicine, Karolinska Institutet, Stockholm, Sweden	Drafting/revision of the article for content, including medical writing for content; study concept or design; and analysis or interpretation of data
Ellen Iacobaeus, MD, PhD	Department of Clinical Neuroscience, Karolinska Institutet; Department of Neurology, Karolinska University Hospital, Stockholm, Sweden	Drafting/revision of the article for content, including medical writing for content; major role in the acquisition of data; study concept or design; and analysis or interpretation of data

## Glossary

AOMS: adult-onset MS
DMT: disease-modifying therapy
EDSS: Expanded Disability Status Scale
HR: hazard ratio
IQR: interquartile range
LOMS: late-onset MS
MS: multiple sclerosis
PPMS: primary progressive MS
RRMS: relapsing-remitting MS
